# Chitosan/Alginate Nanogels Containing Multicore Magnetic Nanoparticles for Delivery of Doxorubicin

**DOI:** 10.3390/pharmaceutics15092194

**Published:** 2023-08-24

**Authors:** Sérgio R. S. Veloso, Eva S. Marta, Pedro V. Rodrigues, Cacilda Moura, Carlos O. Amorim, Vítor S. Amaral, Miguel A. Correa-Duarte, Elisabete M. S. Castanheira

**Affiliations:** 1 Physics Centre of Minho and Porto Universities (CF-UM-UP), Campus de Gualtar, University of Minho, 4710-057 Braga, Portugal; sergioveloso96@gmail.com (S.R.S.V.);; 2 LaPMET Associate Laboratory, Campus de Gualtar, University of Minho, 4710-057 Braga, Portugal; 3 Department of Polymer Engineering, Institute for Polymers and Composites (IPC), University of Minho, 4804-533 Guimarães, Portugal; 4 Physics Department and CICECO, Campus de Santiago, University of Aveiro, 3810-193 Aveiro, Portugal; amorim5@ua.pt (C.O.A.); vamaral@ua.pt (V.S.A.); 5 Centro de Investigación en Nanomateriais e Biomedicina (CINBIO), Universidad de Vigo, 36310 Vigo, Spain

**Keywords:** nanogels, chitosan, alginate, multicore nanoparticles, magnetic hyperthermia, photothermia, controlled release, doxorubicin, cancer therapy, drug delivery

## Abstract

In this study, multicore-like iron oxide (Fe_3_O_4_) and manganese ferrite (MnFe_2_O_4_) nanoparticles were synthesized and combined with nanogels based on chitosan and alginate to obtain a multimodal drug delivery system. The nanoparticles exhibited crystalline structures and displayed sizes of 20 ± 3 nm (Fe_3_O_4_) and 11 ± 2 nm (MnFe_2_O_4_). The Fe_3_O_4_ nanoparticles showed a higher saturation magnetization and heating efficiency compared with the MnFe_2_O_4_ nanoparticles. Functionalization with citrate and bovine serum albumin was found to improve the stability and modified surface properties. The nanoparticles were encapsulated in nanogels, and provided high drug encapsulation efficiencies (~70%) using doxorubicin as a model drug. The nanogels exhibited sustained drug release, with enhanced release under near-infrared (NIR) laser irradiation and acidic pH. The nanogels containing BSA-functionalized nanoparticles displayed improved sustained drug release at physiological pH, and the release kinetics followed a diffusion-controlled mechanism. These results demonstrate the potential of synthesized nanoparticles and nanogels for controlled drug delivery, offering opportunities for targeted and on-demand release in biomedical applications.

## 1. Introduction

Nanogels are nanoscale three-dimensional networks with the ability to adsorb a large amount of water [1]. Typically, these structures range in size from 10 to 200 nm and exhibit a high degree of hydrophilicity, as well as a tuneable size and composition. The small size enables the nanogels to preferentially accumulate in tumors by leveraging the enhanced permeabilization and retention (EPR) effect. Moreover, the nanogels’ biocompatibility, biodegradability, and ability to encapsulate a broad variety of bioactive molecules make them attractive for drug delivery applications [2,3,4,5].

Polysaccharides and proteins are among the most commonly utilized natural polymers for the development of nanogels [1,2,6,7]. For instance, Wang et al. [2] created bovine serum albumin (BSA)@chitosan nanogels capable of encapsulating doxorubicin (DOX) with an entrapment ratio of 46.3%. The system provided sustained drug release and lower cytotoxicity than using DOX alone. In particular, chitosan, a cationic, naturally derived polysaccharide from chitin, has been an attractive choice in the development of nanogels for the delivery of numerous drugs, such as DOX [8,9,10,11,12], curcumin (CUR) [13,14], 5-fluorouracil [15], and paclitaxel [16].

Chitosan particles possess a positively charged surface that can be coated with polyanions, such as alginate, via electrostatic interactions. This has been shown to improve the stability in physiological conditions and reduce the cytotoxicity, which are both required for further progressing to in vitro and/or in vivo studies [6,7,17,18,19].

The addition of magnetic nanoparticles provides the system with theranostic properties, such as magnetic targeting, magnetic hyperthermia, and the ability to serve as contrast agents or magnetic particle imaging tracers for imaging techniques [20,21,22,23,24,25]. Furthermore, the presence of magnetic nanoparticles can synergistically enhance the efficacy of cancer therapy through magnetic hyperthermia and photothermia using near-infrared (NIR) laser light irradiation [26,27,28,29,30]. The photothermia effect of iron oxide nanoparticles through NIR laser irradiation has been successful against difference cancer cell types [30], and the particles are reported to attain a high energy conversion efficiency [31].

The majority of the reported works on magnetic nanogels focus on synthetic polymers [23], since tailoring polymer structure provides more flexibility in achieving the desired properties for biomedical applications. However, the synthetic polymers have limitations regarding the biodegradability, biocompatibility, and toxicity, and some preparation methods lack scalability [32,33,34]. On the other hand, the natural polymers can offer advantages over the synthetic polymers by overcoming the mentioned limitations, besides being obtained from abundant renewable sources and displaying a high binding capacity for various drugs [35,36].

Doxorubicin (DOX) is an anthracycline known to function as a DNA intercalating agent and to inhibit topoisomerase II [37]. This powerful chemotherapy drug is widely used to treat several cancers, including breast, ovary, lung, bladder, and multiple myeloma [38]. However, a significant challenge lies in its lack of specificity, which restricts the administration of higher therapeutic dosages, as it may lead to dose-dependent side effects, such as myelosuppression and cardiotoxicity [38]. To address these limitations, doxorubicin can be encapsulated in nanoformulations, enabling the controlled and targeted delivery to the desired site, resulting in improved therapeutic efficacy and reduced off-target toxicity. In particular, magnetic nanogels have emerged as a promising drug delivery system due to their ability to minimize premature drug release, besides enabling the magnetic targeting and synergistic effect of thermo-chemotherapy [38,39].

**Scheme 1:**
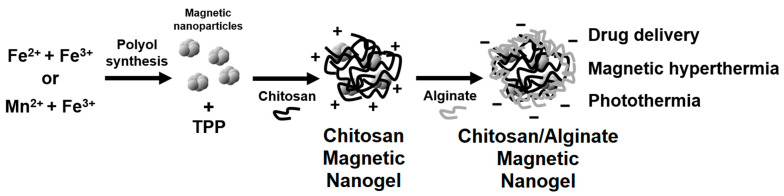
Schematic representation of the developed self-assembled chitosan/alginate magnetic nanogels.

This work aimed to develop self-assembled magnetic nanogels based on alginate and chitosan for the delivery and controlled release of doxorubicin. Firstly, the multicore magnetic nanoparticles of manganese ferrite and magnetite were synthesized and compared, considering the high heating efficiency that can be achieved through photothermia [27,40,41]. The system was prepared through self-assembly using an entirely water-based procedure (Scheme 1). The method consisted in initially dispersing magnetic nanoparticles in a tripolyphosphate (TPP) solution, which is then added to a chitosan solution. Finally, the surface charge of the nanogels is made negative through the addition of an alginate solution. The influence of coating the nanoparticles with citrate was assessed to evaluate the tuneability of the gels’ size and encapsulation efficiency, as a negatively charged surface might favor the adsorption of the positively charged doxorubicin. Additionally, the functionalization with BSA, as a means for assessing the tuneability of the nanoparticles’ size and inclusion of hydrophobic cavities, was also explored. The developed nanogels were tested for the release of doxorubicin in different conditions, and the possibility of using photothermia to enhance its release was also studied.

## 2. Materials and Methods

### 2.1. Synthesis of Nanoparticles and Development of Magnetic Nanogels

#### 2.1.1. Synthesis of Magnetic Nanoparticles (NPs)

Magnetic nanoparticles of magnetite and manganese ferrite were synthesised by a method adapted from Bertuit et al. [40] and Hugounenq et al. [42]. Briefly, FeCl_3_·6H_2_O (2 mmol, 2 equiv) and FeSO_4_·7H_2_O or MnSO_4_·H_2_O (1 mmol, 1 equiv) were dissolved overnight in a mixture of DEG (20 mL) and NMDEA (20 mL) under a N_2_ inert atmosphere. Simultaneously, NaOH pellets (8 mmol, 8 equiv) were ground and dissolved overnight in a mixture of DEG (10 mL) and NMDEA (10 mL). After 30 min under a N_2_ inert atmosphere, the two solutions were mixed via magnetic stirring for 1 h, still under a N_2_ inert atmosphere. A total of 250 μL of ultrapure water was added, and the solution was then heated to 220 °C (reflux), in which the rate between 60 °C and 220 °C was 10 °C/5 min. After a holding temperature of 1 h, the solution was allowed to cool down to room temperature. The suspension was diluted in ethanol and washed two times through centrifugation with ethanol (20 min, 9000 g). The black solid was dried at 100 °C and later redispersed in water.

#### 2.1.2. Citrate Functionalized Magnetic Nanoparticles (NPs@BSA)

Magnetic nanoparticles were functionalized with citrate as described elsewhere [43]. In summary, 50 mg of NPs were dispersed in 10 mL of trisodium citrate 0.3 M at pH = 7, and sonicated for 30 min. Then, the solution was stirred at 80 °C for 2 h, washed with water:acetone 1:1, and stored in water for further use. The synthesized particles’ size was measured through Transmission Electron Microscopy (TEM), and the functionalization was evaluated through Dynamic Light Scattering (DLS) measurements of the hydrodynamic diameter and zeta potential.

#### 2.1.3. Synthesis of Bovine Serum Albumin Functionalized Magnetic Nanoparticles (NPs@BSA)

For the functionalization with BSA, EDC (0.1 mg EDC/mg NP) was initially dissolved in a dispersion of citrate-functionalized magnetic NPs (2 mg/mL) under stirring at room temperature. Then, some amount of BSA was added for the appropriate final concentration (0.8, 2.4, or 4 mg/mL), and the reaction was left stirring for 24 h. The NPs@BSA were separated by centrifugation at 9000 g for 30 min and washed with deionized water. Finally, the nanoparticles were redispersed and stored in water at 4 °C.

#### 2.1.4. Development of Magnetic Nanogels

Magnetic nanogels were developed through a method adapted from Schütz et al. [6]. Briefly, a 0.1 *w*/*v*% chitosan (448869, Sigma-Aldrich, Reykjavik, Iceland, low molecular weight, 75–85% deacetylation) solution was prepared by dissolving chitosan in ultrapure water and gradually adding 1 M HCl until the pH stabilized between 3.5 and 4.0. Subsequently, 0.1 *w*/*v*% tripolyphosphate (TPP) solution was added dropwise to a 9-fold volume of chitosan solution with vigorous stirring. To obtain magnetic nanogels, the nanoparticles were dispersed in the TPP solution for a concentration of 2 mg/mL. The mixture was stirred for at least 1 h. For addition of doxorubicin, a 0.1 *w*/*v*% TPP solution with 200 µM of doxorubicin was employed.

For the surface decoration with alginate (180947, Sigma-Aldrich), the above formulation was diluted 1:1 with ultrapure water. The resulting nanogel dispersion was then added dropwise to a 0.1 *w*/*v*% sodium alginate solution in a ratio 1:1 under strong agitation. The pH was maintained between 7.1 and 7.5 by adding 0.025 M NaOH. Afterward, the dispersion was washed at 3500 g for 10 min with deionized water and finally stored in 500 µL of water at 4 °C.

### 2.2. Characterization Techniques

#### 2.2.1. General Spectroscopic Methods

Absorption spectra were recorded using a Shimadzu UV-3600 Plus UV-Vis-NIR spectrophotometer (Shimadzu Corporation, Kyoto, Japan). X-ray diffraction (XRD) analyses were conducted with a conventional PANalytical X’Pert PRO diffractometer (Malvern Panalytical Ltd., Malvern, UK), utilizing Cu K_α_ radiation, in a Bragg–Brentano configuration at the Electron Microscopy Unit, University of Trás-os-Montes and Alto Douro (UTAD), Vila Real, Portugal. Raman spectroscopy measurements were performed at room temperature using a LabRAM HR Evolution confocal microscope system (HORIBA France SAS, Palaiseau, France), equipped with a high-resolution grating of 1800 grooves mm^−1^. The excitation line, 532 nm, of an NIR diode laser was focused onto the sample by a ×50 objective with a numerical aperture (NA) value of 0.80 in a backscattering geometry. The spectra were acquired with a measured power of about 1.43 mW on the sample, with a spectral acquisition time of 5 s averaged over 10 accumulations and the range 100–1000 cm^−1^. The average hydrodynamic diameter and zeta potential of the nanoparticles (n = 3 independent runs) were measured in phosphate buffer pH 7.4 at 0.01 mg/mL in a Dynamic Light Scattering (DLS) equipment Litesizer^TM^ 500 from Anton Paar (Anton Paar GmbH, Graz, Austria), using a semiconductor laser diode of 40 mW and λ = 658 nm, a backscatter angle (175°), and a controlled temperature of 25 °C.

#### 2.2.2. Transmission Electron Microscopy (TEM)

Nanoparticles’ TEM images were captured using a high contrast JEOL JEM-1010, operating at 100 kV (CACTI, Vigo, Spain). For the STEM images, a NanoSEM—FEI Nova 200 was used, operating at 15 kV, coupled to an Electron Dispersive Spectroscopic analyzer (EDS) and Electron Backscatter Diffraction EDAX—Pegasus X4M analyser and detection system (EBSD) at SEMAT/UM (Serviços de Caracterização de Materiais, Guimarães, Portugal). A small portion of the sample was placed onto a TEM 400 mesh copper grid with Formvar/Carbon (ref. S162-4 from Agar Scientific, London, UK), held by tweezers, and the excess solution was cleaned. The processing of STEM images was performed using ImageJ software (National Institutes of Health, NIH, Bethesda, MD, USA, version 1.53t), which consisted of enhancing local contrast and adjusting brightness followed by manual selection of nanoparticles/nanogels.

#### 2.2.3. Magnetic Properties

Magnetic measurements were performed in an MPMS3 SQUID magnetometer (Quantum Design Inc., San Diego, CA, USA). The field-dependent magnetization (hysteresis cycles) of the samples were measured in the large field range (up to H = 5570.42 kA/m or B = 7 T) for each sample. In all the cases at 300 K, given the room temperature applications they are designed for, a specific magnetic field correction for the trapped flux in the superconducting coil was conducted, achieving an accuracy of residual less than 0.16 kA/m [44].

#### 2.2.4. Magnetic Hyperthermia Measurements

With the aim of evaluating the heating performance, magneto-caloric measurements were carried out using a hyperthermia system magneTherm (nanoTherics, Warrington, UK), working at f ≈ (162, 271, 383, 617) kHz and at the magnetic field H = (13.56, 12.76, 9.57) kA/m. For all experiments, the initial temperature was stabilized before starting the measurement. Next, the AC magnetic field was applied for 10 min, and the temperature variation was recorded using a thermocouple.

A phenomenological Box–Lucas equation was fitted to the temperature variation over time ($\Delta T\left(t\right)$) profiles during exposure to the alternating magnetic field. The equation is described by the parameter A (saturation temperature) and B (related with the curvature of the profile) as follows:

(1) $$\Delta T\left(t\right)=A(1-e^{-Bt})$$

The product A⨯B at t=0 is equivalent to the ratio of the initial slope of the temperature curves, dT/dt, required to calculate *SLP*, which is defined as follows:

(2) $$SLP=\frac{m_{sample}c}{m_{NP}}\frac{dT}{dt}$$

where $m_{sample}$ is the total mass of the sample (g), *c* is the specific heat capacity of the sample (approximated to $c_{water}$ = 4.185 J/g/K), and $m_{NP}$ is the total mass of nanoparticles in the sample (g).

#### 2.2.5. Photothermia Measurements

To obtain the photothermia profiles upon irradiation of the nanoparticles, the sample (2 mL) was prepared in a 1 cm polystyrene cuvette, placed in a sample cuvette holder with the temperature stabilized at 37 °C, and irradiated with an 808 nm laser (1 W/cm^2^). The temperature was measured by placing a K-type thermocouple 1 cm inside the sample.

The light-to-heat conversion efficiency was calculated from the equation [45]:

(3) $$\eta =B\cdot \Delta T\cdot \frac{m_{sample}c}{P_{0}(1-10^{-A})}$$

where ΔT is the temperature variation, $m_{sample}$ is the total mass of the sample, c is the specific heat capacity of the sample (approximated to $c_{water}$ = 4.185 J/g/K), $P_{0}$ is the incident laser power, A is the sample absorbance, and B is the constant rate of heat dissipation from the solution to the external environment, which is measured during heat relaxation as $e^{-Bt}=\frac{T\left(t\right)-T_{0}}{T_{m}-T_{0}}$, in which $T\left(t\right)$ is the temperature measured at time t, $T_{0}$ is the starting temperature, and $T_{m}$ is the maximum temperature. The value of B was determined to be 0.0035 ± 0.0002 s^−1^.

### 2.3. Swelling Assays

The nanogels were subjected to swelling experiments by immersing them in phosphate buffer 0.1 M pH = 7.4 for 72 h. At specific time intervals, the nanogels were centrifuged and weighed (*W_s_*). Afterward, the nanogels were dried and weighed again (*W*_0_). To determine the swelling ratio, the following equation was used:

(4) $$SR=\frac{\left(W_{s}-W_{0}\right)}{W_{0}}$$

### 2.4. Doxorubicin Loading and Release Assays

Nanogels loaded with doxorubicin were prepared by initially employing a 0.1 *w*/*v*% TPP solution containing 200 µM of doxorubicin. The supernatant obtained from the centrifugation of the nanogels was stored and its fluorescence measured to determine the non-encapsulated doxorubicin. The encapsulation efficiency was determined as follows:

(5) $$EE\left(\%\right)=\frac{\left({amount}_{total}-{amount}_{non-encapsulated}\right)}{{amount}_{total}}\times 100$$

For the release assays, 100 µL of the nanogels dispersion (500 µL at a nanogel concentration of ~6 mg/mL) were added to microcentrifugal tubes. Then, phosphate buffer 0.1 M pH = 7.4 or 6 (700 µL) was added to keep pH constant; an Amicon^®^ Ultra-0.5 mL centrifugal filter with 0.1 μm pore size was immersed and was filled with 200 µL of buffer (top reservoir). Aliquots were taken and replaced with fresh buffer, and fluorescence (*λ_exc_* = 480 nm) was measured to determine the concentration at each time point. For the assays with laser irradiation at 808 nm (1 W/cm^2^), the samples were irradiation for 10 min after each aliquot. Release profile assays were performed in triplicate.

## 3. Results and Discussion

### 3.1. Characterization of the Magnetic Nanoparticles

**Figure 1:**
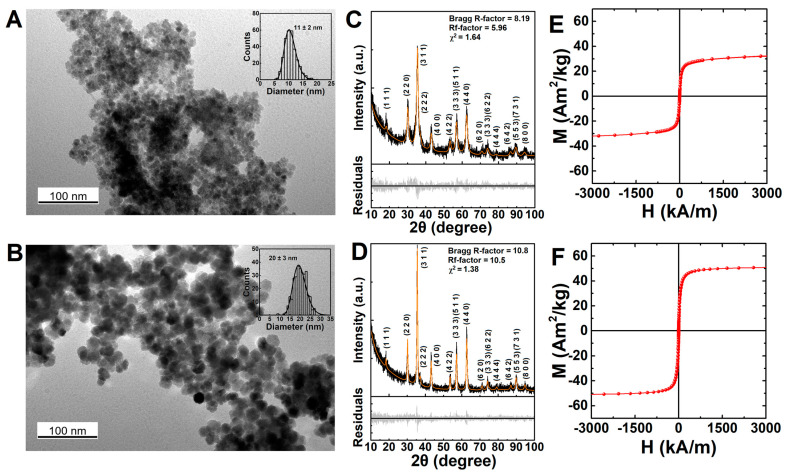
TEM images of the (**A**) MnFe_2_O_4_ nanoparticles and (**B**) Fe_3_O_4_ nanoparticles. Inset: Histograms of the particle sizes measured in the respective samples. XRD diffraction pattern and fitted Rietveld refinement pattern of the (**C**) MnFe_2_O_4_ and (**D**) Fe_3_O_4_ nanoparticles. Magnetization dependence on the applied magnetic field measured at 300 K for (**E**) MnFe_2_O_4_ nanoparticles and (**F**) Fe_3_O_4_ nanoparticles.

The synthesized nanoparticles displayed a flower-like morphology, which is in line with the morphologies obtained for iron oxide particles through the employed polyol synthesis [40,46,47]. The iron oxide and manganese ferrite nanoparticles were obtained with an average physical diameter of 20 ± 3 nm and 11 ± 2 nm (Figure 1A,B and Figure S1), respectively, and with low size polydispersity, as determined from the analysis of TEM images (N = 300). The size of the magnetite nanoparticles was in line with other reported sizes of multicore particles obtained through the same method [40,42,47], while the use of Mn(II) resulted in smaller particle sizes.

The X-ray diffraction (XRD) patterns in Figure 1C,D suggested the crystalline nature of the synthesized nanoparticles and displayed the Bragg’s reflections characteristic of the Fd-3m space group, as highlighted in the diffractogram [48,49]. Rietveld analysis was performed using a phase adapted from a CIF file of iron oxide (CIF file 2300618, space group Fd-3m). The refinement resulted in a good quality fitting, as indicated from the parameters Rf and χ^2^ included in Figure 1C,D. An average crystallite size of 6 nm and 14 nm was obtained for manganese ferrite and magnetite nanoparticles, respectively. The smaller crystalline size obtained from XRD can be associated with the misalignment between the grains that arise from crystalline defaults at the grain interfaces [42]. Additionally, the lattice parameter of 8.413 Å was calculated for the manganese ferrites, and 8.381 Å for the magnetite nanoparticles, which are in agreement with the commonly reported values [40,48,50,51,52]. The magnetite nanoparticles were also confirmed to not be oxidized, as suggested by the presence of a band above 1000 nm, associated with the intervalence charge transfer between Fe^2+^ and Fe^3+^ in magnetite (Figure S2) [53].

Regarding the magnetic properties, the magnetite nanoparticles (51.1 Am^2^/kg) displayed larger saturation magnetization than the manganese ferrite (33.8 Am^2^/kg), thus indicating the detrimental effect of replacing Fe(II) with Mn(II) in the employed synthesis method. Nonetheless, both displayed an M_r_/M_s_ < 0.1 (Table S1), which suggests that both nanoparticles are superparamagnetic [54].

In solution, the manganese ferrite nanoparticles’ larger hydrodynamic diameter (247 ± 15 nm) compared with the magnetite nanoparticles’ (148 ± 1 nm) may be associated with the smaller zeta potential (<30 mV) leading to aggregation, which is also suggested by a larger polydispersity index (Figure S3 and Table S2).

### 3.2. Assessment of the Magnetic Nanoparticles for Photothermia

**Figure 2:**
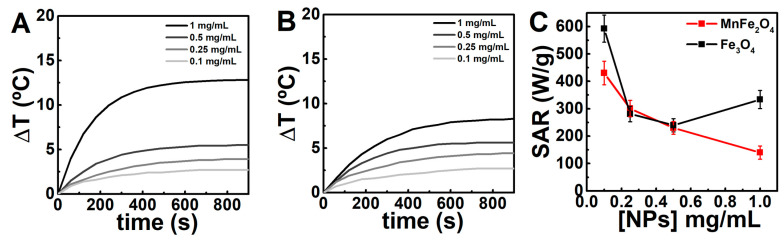
Temperature variation profiles of different concentrations of the (**A**) Fe_3_O_4_ and (**B**) MnFe_2_O_4_ nanoparticles upon laser irradiation at 808 nm with power density of 1 W/cm^2^. (**C**) Dependence of the specific absorption rate (SAR) on the nanoparticle concentration.

Both MnFe_2_O_4_ and Fe_3_O_4_ nanoparticles displayed high heating generation upon laser irradiation at 808 nm (1 W/cm^2^), as displayed in Figure 2. Notably, the Fe_3_O_4_ nanoparticles could induce a temperature change of ~12 °C in 6 min at a concentration of 1 mg/mL. This strong enhancement in temperature variation can be associated with the clustering of the nanoparticles at high concentrations [31,55].

The stronger heating efficiency of the Fe_3_O_4_ nanoparticles is also indicated by the calculated specific absorption rate (SAR), which displayed a value of ~600 W/g at 0.1 mg/mL. The high heating efficiency might result from the larger mean volume of the Fe_3_O_4_ nanoparticles and the lower concentration of defects [40]. Regarding the latter, an Urbach energy (E_U_) of 1.3 eV and 1.1 eV was obtained for the Fe_3_O_4_ and MnFe_2_O_4_ nanoparticles, respectively (Figure S4). Smaller E_U_ values correspond to high-defect systems, and consequently, are associated with a decrease in heating efficiency [40]. However, the SAR values can also be linked to the mean hydrodynamic size [56], as the concentration affects the laser beam diffusion. In this sense, the lower mean hydrodynamic size of the Fe_3_O_4_ nanoparticles might contribute to its higher SAR value, compared with the MnFe_2_O_4_ nanoparticles. In addition, as reported in other studies [41,45,55], the SAR value decreases with the nanoparticle content as a result of the light absorption of the colloidal suspension. This can be understood in terms of the Lambert–Beer’s law, i.e., the unequal distribution of the incident light, which is mainly absorbed and scattered in the front of the cuvette, leads to a gradient in the incident power [31]. Hence, as the light absorption is not homogenous along the nanoparticle’s suspension, the heat release will also be inhomogeneous. Additionally, a photothermal conversion efficiency (η) of 33 ± 4% and 20 ± 3% was obtained for the Fe_3_O_4_ and MnFe_2_O_4_ nanoparticles, respectively, which are comparable to or overcome other reported nanoparticles [31,41,45,55]. In particular, the obtained value for the Fe_3_O_4_ nanoparticles is in line with the commonly reported heating efficiencies for iron-based nanomaterials [40,55,57]. The magnetic hyperthermia was also assessed (Figure S5), but the heating efficiency was lower than photothermia, as described in other works [31,45].

### 3.3. Functionalization of the Nanoparticles

In this work, the functionalization with citrate and BSA was studied to understand if a small negatively charged molecule or additional hydrophobic cavities [58,59,60], respectively, could improve the drug encapsulation. Additionally, the encapsulation of proteins, namely with biological activity, can be of potential interest for biomedical applications. The coating with citrate resulted in a decrease in the hydrodynamic diameter, a negative zeta potential (around −20 mV) at pH = 7.4, and a polydispersity index (PDI) within the 0.1–0.2 range, which is indicative of good stability and less aggregation compared with the uncoated nanoparticles. For simplicity, the remaining studies with BSA were carried out with one of the magnetic nanoparticles (Fe_3_O_4_). The functionalization with BSA led to an incremental increase in the hydrodynamic size, a low PDI, and a largely unchanged zeta potential across the different preparations but was less negative than the citrate-stabilized nanoparticles. The correlograms and UV-vis absorption spectra are included in the Supplementary Information (Figures S6 and S7).

**Figure 3:**
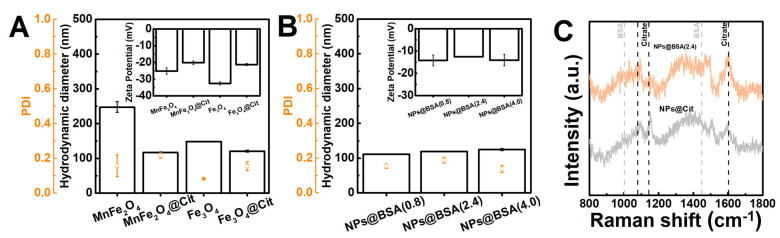
Hydrodynamic diameter (bars) and polydispersity index (PDI, squares) of the (**A**) bare and the citrate-stabilized nanoparticles, and (**B**) of the BSA-functionalized Fe_3_O_4_ nanoparticles. Insets: zeta potential of the respective nanoparticles. The particles functionalized with BSA were labelled NPs@BSA(x) (where x = 0.8, 2.4, and 4.0), corresponding to reaction with 0.8, 2.4, and 4 mg/mL of BSA. (**C**) Raman spectra of Fe_3_O_4_ nanoparticles functionalized with citrate and BSA.

The absorption spectra profiles of the nanoparticles were similar, in which the different intensities could be related with the particle size (Figure S7). In Figure 3C, commonly reported Raman shifts for citrate [61,62,63,64,65] and BSA [66,67] are highlighted, which further confirms the coupling of citrate and BSA to the particles. The spectrum of citrate-functionalized nanoparticles showed modes near 1080 cm^−1^ (νCC symmetric), 1190 cm^−1^ (νCCO), and 1610 cm^−1^ (νCO^2−^ asymmetric), as well as a peak near 1500 cm^−1^ that could be associated with the stretching vibration mode of the carboxylate groups. The presence of BSA resulted in additional peaks, mainly a mode of about 1450 cm^−1^ that can be assigned to the δCH_2_.

### 3.4. Characterization of the Nanogels

Nanogels were prepared for all particle formulations, including neat nanogels (i.e., nano-hydrogels, NHG). Overall, the resulting nanogels displayed a good polydispersity, a strongly negative zeta potential in pH = 7.4 0.1 M phosphate buffer (around −25 mV), and a size larger than 200 nm, which is consistent with the results reported in [6]. In particular, the polydispersity index (PDI) remained within the range of 0.1–0.2, which is considered an acceptable value for drug delivery applications [68]. As alginate consists of alternating blocks of β-(1→4)-linked-D-mannuronic acid and α-(1→4)-linked-L-glucuronic acid [35], the nanogels are endowed with a negative surface charge.

Noticeably, the BSA-functionalized nanoparticles led to nanogels with a slightly larger hydrodynamic diameter, which can result from the aggregation of these nanoparticles during the preparation, as the zeta potential was lower than the other particles. Additionally, the functionalization with citrate resulted in nanogels with a hydrodynamic diameter similar to the nanogels and smaller than when bare nanoparticles are employed, which might result from the favourable electrostatic interactions between the surface citrate carboxyl moieties and the chitosan positively charged amine groups. A smaller size was also obtained for nanogels with citrate-stabilized MnFe_2_O_4_ nanoparticles (Figure S8).

**Figure 4:**
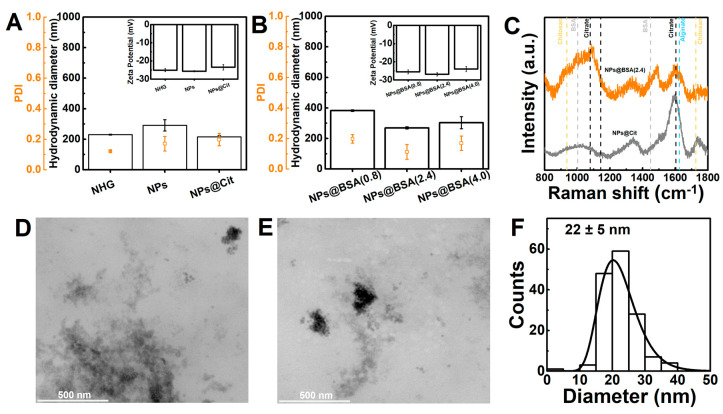
Hydrodynamic diameter (bars) and polydispersity index (PDI, squares) of the (**A**) nanogels prepared with bare and the citrate-stabilized Fe_3_O_4_ nanoparticles, and (**B**) of the BSA-functionalized nanoparticles. Insets: zeta potential of the respective nanogels. (**C**) Raman spectra of nanogels containing Fe_3_O_4_ nanoparticles functionalized with citrate and BSA. (**D**,**E**) TEM images of the nanogels containing citrate-stabilized Fe_3_O_4_ nanoparticles, and (**F**) the respective histogram of the measured particle size.

The encapsulation of nanoparticles into nanogels was also evaluated through Raman spectroscopy. In Figure 4C, commonly reported Raman shifts for chitosan [69] and alginate [70,71] are highlighted, as well as the mentioned BSA and citrate Raman shifts. The nanogels displayed some peaks indicative of the nanoparticles being covered with the polysaccharides, such as a strong broad peak around 1600 cm^−1^ that includes contributions from δNH_2_ and νCO_2_^−^ modes, as well as the vibrational modes of the remaining components (BSA and/or citrate).

Regarding the swelling capability, all the nanogel formulations exhibited fast swelling within the first 3 h, reaching equilibrium after 4 h (Figure S9). The swelling curves could be described by a second-order kinetic model [72], and the fitting of Korsmeyer– Peppas power law suggested that the water transport is driven by a diffusion-control mechanism (Table S3). Remarkably, the magnetic gels demonstrated significantly greater swallowing than the nano-hydrogel. Thus, the larger swallowing might be associated with the increase in hydrophilic groups in the gel matrix present in the surface of the nanoparticles and/or the interaction with the gel network leading to a less dense network [73].

The TEM images of the nanogels containing citrate-stabilized Fe_3_O_4_ (Figure 4D,E) revealed the presence of rounded particles that display some agglomeration, which might result from the drying process of the samples or nanogels containing clusters of nanoparticles. In addition, both TEM and SEM images suggested that the gels are non-porous (Figures S10 and S11). Taking into account the application of the nanogels in drug delivery, non-porous gels can provide a slower drug release rate [74], which is valuable in obtaining stimuli-responsive systems that enable both the controlled and sustained release of the encapsulated drugs. The measurement of the particles revealed an average size of 22 ± 5 nm for the smaller particles and 112 ± 42 nm for the agglomerates (Figure S10), which indicate that the nanogel network might collapse during the preparation of the samples and thus, resulting in a smaller size than the sizes obtained from DLS. Additionally, the results suggest the existence of nanogels containing clusters of magnetic nanoparticles and/or single nanoparticles. These larger nanogel structures were also observed through SEM (Figure S11). Nonetheless, considering the low polydispersity of the samples in DLS and a strongly negative zeta potential, the aggregation of nanogels is likely to be averted in solution.

### 3.5. Drug-Loading and Release Assays

**Figure 5:**
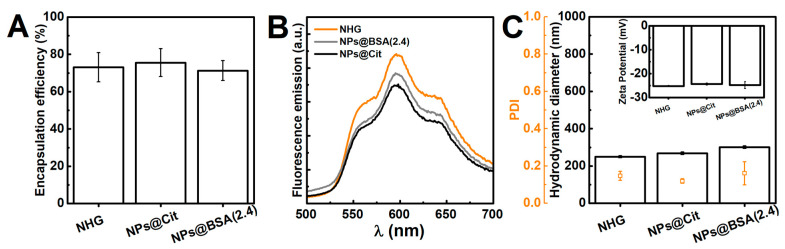
(**A**) Encapsulation efficiency of doxorubicin in nano-hydrogels (NHG), and magnetic nanogels containing citrate- and BSA-functionalized nanoparticles. The nanogels were initially loaded with doxorubicin for a final concentration of 40 µM. (**B**) Fluorescence emission of the doxorubicin loaded nanogels (λ_480nm_ = 480 nm). (**C**) Hydrodynamic diameter and polydispersity index (PDI) of the assessed (magnetic) nanogels loaded with doxorubicin. Insets: zeta potential of the doxorubicin loaded (magnetic) nanogels.

The encapsulation of doxorubicin in biopolymer-based nanogels has been demonstrated to be effective in improving the drug half-life owing to the reduction in nanoparticles’ clearance and by providing stealth properties [38]. Considering the similarity of the results obtained with both magnetic nanoparticles, the drug loading and release assays were tested for the particles with the best hyperthermia properties, the Fe_3_O_4_ nanoparticles. The doxorubicin encapsulation efficiencies (EE%) were determined for nano-hydrogels, and magnetic nanogels containing citrate- and BSA-functionalized Fe_3_O_4_ nanoparticles. As displayed in Figure 5, the encapsulation efficiencies were similar among the nanogels formulations at around 70%, thus indicating that the nanoparticles had little influence on the encapsulation of doxorubicin.

The photophysical properties of doxorubicin allowed for the confirmation of its encapsulation in the nanogels. The strong fluorescence emission, the maximum fluorescence wavelength around 595 nm, and the ratio between the intensities at 550 nm (I_1_) and 595 nm (I_2_) are indicative of the localization of doxorubicin in an environment less polar than water [48,75]. According to the intensity ratios (I_1_/I_2_ ~0.543 ± 0.014) described by Karukstis et al. [75], the polarity is similar to 1-hexanol (dieletric constant, ε = 13.3). The shape of the emission band also approaches the doxorubicin spectra described by Cardoso et al. [76] in organic protic solvents, such as ethanol and methanol. A fluorescence emission quenching effect was also observed for the magnetic nanoparticles, which is stronger for the gels containing citrate-stabilized nanoparticles. This effect has been reported for several antitumour drugs encapsulated in magnetic gels [48,77], which is indicative of the close proximity of the drugs to the nanoparticles. Hence, considering the mentioned results, doxorubicin might localize close to the core of the nanogels and establish hydrogen bonds with the hydroxyl groups of the polysaccharide chains. In addition, the encapsulation of doxorubicin did not induce major changes in the hydrodynamic diameter and polydispersity, and as the zeta potential remained mostly unaffected (correlograms in Figure S12), it further suggests doxorubicin localization in the core of the nanogels. Nonetheless, the formation of complexes between doxorubicin and alginate is not excluded. It is also worth noting that the hydrodynamic size of doxorubicin-loaded magnetic nanogels (~200 nm) and their zeta potential (~−25 mV) endow the nanosystem as suitable for intravenous injection [78,79].

**Figure 6:**
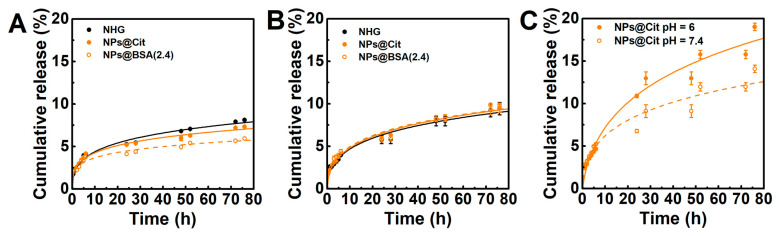
Cumulative doxorubicin release profiles from nano-hydrogels (NHG) and magnetic nanogels containing citrate- and BSA-functionalized nanoparticles at (**A**) pH = 7.4 and (**B**) pH = 6, and (**C**) from magnetic nanogels with citrate-functionalized nanoparticles exposed to 808 nm laser irradiation (1 W/cm^2^, 10 min after each aliquot). The release profiles were fitted to Gompertz model over the 76 h.

The release of doxorubicin from nanogels was carried out at pH = 6 and pH = 7.4 to assess the effect of pH and simulate the acidic conditions in tumor microenvironments and neutral physiological media [80,81], respectively. In addition, the particle functionalization (citrate or BSA) was also evaluated and compared with the release from nanogels. The passive release profiles depicted in Figure 6A,B demonstrated an initial fast release rate of doxorubicin during the first 24 h, which can be associated to the loosely bound doxorubicin on the nanogels. This initial phase was followed by a slower release in the subsequent hours. Similar release profiles have been observed for other types of self-assembled (nano)gels [82,83,84]. Importantly, the drug is not completely released after 3 days, indicating a strong association between doxorubicin and the nanogel matrix. A comparison of the total drug release at pH = 7.4 and 6 demonstrates a higher release of doxorubicin at lower pH. However, it is worth noting that the observed enhancement in drug release is not strongly different during the initial 24 h. This phenomenon can be attributed to the increased stability of the nanogels’ matrix resulting from enhanced electrostatic interactions between alginate and chitosan, which might effectively slow down the release of the drug [85]. Nevertheless, the results are in line with the increased hydrophilicity of doxorubicin at an acidic pH facilitating its release. Additionally, the incorporation of nanoparticles contributed to an improved sustained release of doxorubicin, namely in the gels with BSA-functionalized nanoparticles. This effect can be associated with the additional hydrophilic and hydrophobic contacts provided by BSA [60]. BSA-based nanoparticles have been demonstrated to be a suitable strategy to obtain pH-sensitive systems for the release of doxorubicin [86]. Therefore, the results indicate that the functionalization with BSA provides a suitable strategy to further reduce the release of doxorubicin in physiological pH while guaranteeing an unaffected enhanced release at pH 6. Notably, the release profiles indicate that the developed magnetic nanogels display improved sustained doxorubicin release compared with other reported nanogels based on chitosan and/or alginate [87,88,89]. In addition, the developed strategy might be adaptable to the encapsulation of proteins with biological functions, such as lactoferrin [90].

The dose-dependent cardiotoxicity of doxorubicin is a significant limitation, frequently resulting in cardiac atrophy years after the anthracycline exposure, which has been demonstrated in mice to be a dose-dependent effect that begins at relatively low dose exposure [91]. Hence, considering the need for a better control of drug release to minimize the side effects and increase the efficacy of the therapeutic strategy, the nanogels containing citrate-stabilized nanoparticles were evaluated for their ability to release drugs upon laser irradiation. The results showed that irradiation with an 808 nm laser (1 W/cm^2^) led to a slight enhancement of the release within the initial 24 h, and also enhanced the release during the subsequent slower phase after each irradiation period. Notably, this effect was observed at both pH 7.4 and pH 6, suggesting that the systems are not only pH-responsive but also temperature-responsive. Additionally, in line with the passive release results, active release was also more pronounced at pH 6 compared with pH 7.4. This finding indicates that the effect will be even stronger in proximity to the tumor, as tumors typically exhibit a more acidic pH in their microenvironment. Consequently, drug release can be spatially enhanced and controlled within tumors. Considering that controlled release systems aim to maintain drugs at target sites with therapeutic concentrations, as well as regulate the release rate and duration, the developed magnetic nanogels hold promise as nanosystems for delivering doxorubicin.

The cumulative drug release profiles were subjected to fitting with various mathematical models (Table S4), including the Korsmeyer–Peppas and first-order models [92]. The observed curvilinear nature of the release profiles suggests that the nanoparticles deviate from following a first-order kinetic pattern. Instead, the fitting analysis of the release profiles revealed that the Korsmeyer–Peppas model provide a better description of the results. This model can be applied to systems with different geometries, as long as perfect sink conditions are maintained [92,93]. Furthermore, the Gompertz empirical model also demonstrated a good fit to the data, allowing the comparison of the release kinetics.

The parameter n in the Korsmeyer–Peppas model serves as an indicator of the diffusion mechanism (Table S5). Values of n below 0.5 are associated with a diffusion-controlled release mechanism, which was obtained during the initial 6 h of analysis (Table S6). This further supports the stability of the nanosystems and suggests that a Fickian diffusion process governs the sustained release of doxorubicin. Interestingly, the diffusion mechanism remained unchanged during laser irradiation, with the heating effect originating from the magnetic nanoparticles primarily manifesting as an increase in the rate constant. This observation indicates that the system maintains its stability during irradiation, as the release mechanism remains consistent, and the release is potentially enhanced due to the thermal effects facilitating drug diffusion.

It is also important to note that chitosan/alginate nanogels have been widely reported to be biocompatible and suitable for cancer therapy [79]. For example, Yoncheva et al. [78] developed chitosan/alginate nanogels with an approximate diameter around 300 nm, which displayed a doxorubicin release profile characterized by pH-responsive behavior. The authors reported the nanogels’ efficacy against melanoma in in vivo experiments, revealing suitable intracellular accumulation and a prolonged cytotoxic impact on cancer cells. However, composite-based nanogels can provide further improved therapeutic outcomes by enabling the use of dual strategies, such as pH/redox, magnetic/pH, and photothermal responsiveness [38,94]. For instance, Bergueiro et al. [95] developed nanogels composed of a thermoresponsive polymer cross-linked by plasmonic gold nanoparticles, showcasing a temperature-triggered chemotherapy approach that effectively inhibited tumour growth. However, the system displayed a burst release in the initial 6 h, which raised concerns regarding undesired side effects, it and could not be magnetically guided. In contrast, Huang et al. [96] developed GSH/pH co-triggered magnetic nanogels based on thiolated alginate and thiolated magnetic nanoparticles, which was found to have cytocompatibility and also to be effective in inhibiting tumor cell growth. Recent studies have demonstrated the superior photothermia capability of iron oxide nanoparticles compared with magnetic hyperthermia, particularly achieving large heating efficiency at a low dosage of nanoparticles [31,97,98]. Hence, in this work, besides the sustained release and pH-responsiveness achieved with the chitosan/alginate nanogels, the use of the photothermia capability of magnetic nanoparticles can provide a valuable strategy in controlling drug release, as in the case of gold nanoparticles, while enabling the use of magnetic targeting and synergistically enhancing the therapeutic efficacy.

## 4. Conclusions

In this work, superparamagnetic iron oxide (Fe_3_O_4_) and manganese ferrite (MnFe_2_O_4_) nanoparticles with multicore-like morphology and an average physical diameter of 20 ± 3 nm and 11 ± 2 nm were synthesized, respectively. The Fe_3_O_4_ nanoparticles showed larger saturation magnetization, magnetic hyperthermia, and photothermia (at 808 nm) heating efficiency compared with manganese ferrite nanoparticles. The functionalization of the nanoparticles with citrate and bovine serum albumin (BSA) resulted in changes in their hydrodynamic properties and stability, and both could be formulated into nanogels based on chitosan and alginate using a self-assembly method. The obtained magnetic nanogels were negatively charged (~−25 mV), with hydrodynamic diameters in the 200–300 nm range, and displayed a similar doxorubicin encapsulation efficiency, which was determined to be around 70%. The release of doxorubicin from the nanogels exhibited a sustained release profile, driven by a diffusion-controlled mechanism. The incorporation of nanoparticles, namely those functionalized with BSA, improved the sustained release at physiological pH. Additionally, the acidic pH and photothermia effect enhanced the release of doxorubicin from the magnetic nanogels. Overall, the developed magnetic nanogels displayed promising results for controlled and enhanced drug delivery, with potential applications in cancer therapy.

## Data Availability

The data presented in this study are available in this article (and Supplementary Materials).

## Supplementary Materials

The following supporting information can be downloaded at: https://www.mdpi.com/article/10.3390/pharmaceutics15092194/s1, Figure S1: TEM images of nanoparticles; Figure S2: UV-vis spectra of nanoparticles; Table S1: Magnetic properties of nanoparticles; Figure S3: DLS results of nanoparticles; Table S2: Hydrodynamic properties of nanoparticles; Figure S4: Urbach plots of the nanoparticles; Figure S5: Heating profiles obtained from magnetic hyperthermia; Figure S6: DLS results of functionalized nanoparticles; Figure S7: UV-vis results of functionalized nanoparticles; Figure S8: DLS results of nanogels; Figure S9: Swelling kinetics of nanogels; Figure S10: TEM results of nanogels; Figure S11: SEM images of nanogels; Figure S12: DLS results of magnetic nanogels loaded with doxorubicin; Table S3: Parameters obtained from the fitting of Korsmeyer–Peppas and second-order swelling kinetics models to the swelling curves; Table S4: Coefficients of determination of several models fitted to release profiles; Table S5: Release coefficients obtained for the Korsmeyer–Peppas and Gompertz models fitted to the release profiles.
