# Supplementary material for: Chitosan/Alginate Nanogels Containing Multicore Magnetic Nanoparticles for Delivery of Doxorubicin
Source: Pharmaceutics. 2023 Aug 24;15(9):2194. doi: 10.3390/pharmaceutics15092194 (PMC10538132; doi:10.3390/pharmaceutics15092194)
Supplement: Supplementary file 1 [file pharmaceutics-15-02194-s001.zip › pharmaceutics-2537383-supplementary.pdf]

Chitosan/alginate nanogels containing multicore magnetic nanoparticles for delivery of doxorubicin

Sérgio R. S. Veloso[1,2], Eva S. Marta[1,2], Pedro V. Rodrigues[3], Cacilda Moura[1,2], Carlos O. Amorim[4], Vítor S. Amaral[4], Miguel A. Correa-Duarte[5], Elisabete M. S. Castanheira[1,2,*]

[1] Physics Centre of Minho and Porto Universities (CF-UM-UP), University of Minho, Campus de Gualtar, 4710-057 Braga, Portugal.
[2] LaPMET Associate Laboratory, University of Minho, Campus de Gualtar, 4710-057 Braga, Portugal.
[3] Institute for Polymers and Composites (IPC), Department of Polymer Engineering, University of Minho, 4804-533 Guimarães, Portugal
[4] Physics Department and CICECO, University of Aveiro, Campus de Santiago, 3810-193 Aveiro, Portugal.
[5] Centro de Investigación en Nanomateriais e Biomedicina (CINBIO), Universidad de Vigo, 36310 Vigo, Spain.

* Correspondence: ecoutinho@fisica.uminho.pt; Tel.: +351-253-604-321;

Supplementary Material

Characterization of the magnetic nanoparticles

A B

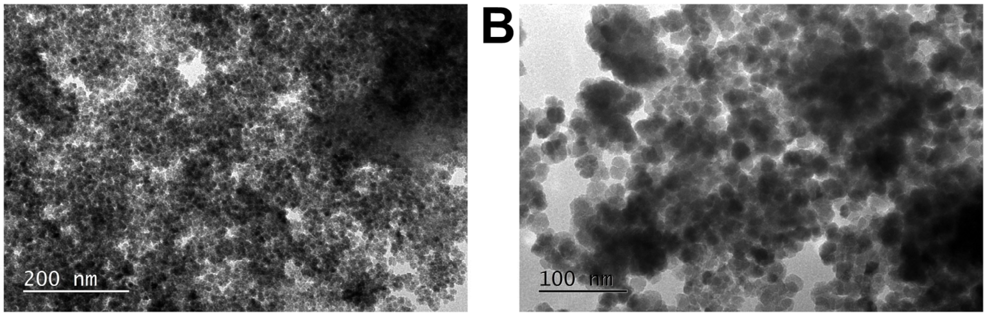

Figure S1. TEM images of the (A) $MnFe_2O_4$ and (B) Fe_3O_4 nanoparticles.

The band-gap energy (E_g) of the semiconductor nanoparticles can be calculated from the Tauc plot:

$$(\alpha(\lambda) \cdot \mathrm{E}(\lambda))^n \propto (\mathrm{E}(\lambda) - E_g) \quad \text{(S1)}$$

where $\alpha(\lambda) = \mathrm{A}(\lambda)/l$ is the absorption coefficient in cm^{-1}, n is an exponent that depends on the nature of the transition, $\mathrm{E}(\lambda) = hc/\lambda$ is the incident photon energy, and E_g is the optical band gap. Iron oxide nanoparticles are reported to be direct and allowed bang-gap semiconductors, i.e. $n = 1/2$ [1]. The interpolation of the linear part of the curve to the energy axis allows the determination of the band gap.

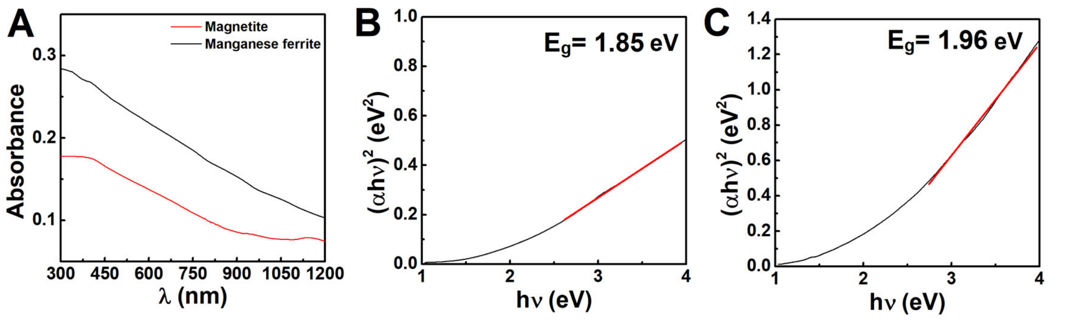

Figure S2. (A) UV-vis spectra of the Fe_3O_4 and $MnFe_2O_4$ nanoparticles and (B,C) the respective Tauc plots (B: Fe_3O_4; C: $MnFe_2O_4$).

Table S1. Experimentally determined magnetic properties measured at 300 K: saturation magnetization (M_S); remnant magnetization (M_R); squareness (M_R/M_S); coercivity (H_C).

Nanoparticle	T (K)	M_S (Am^2/kg)	H_C (kA/m)	M_R (Am^2/kg)	M_R/M_S
Fe_3O_4	300	51.1	2.1	2.5	0.05
$MnFe_2O_4$	300	33.8	0.8	0.5	0.01

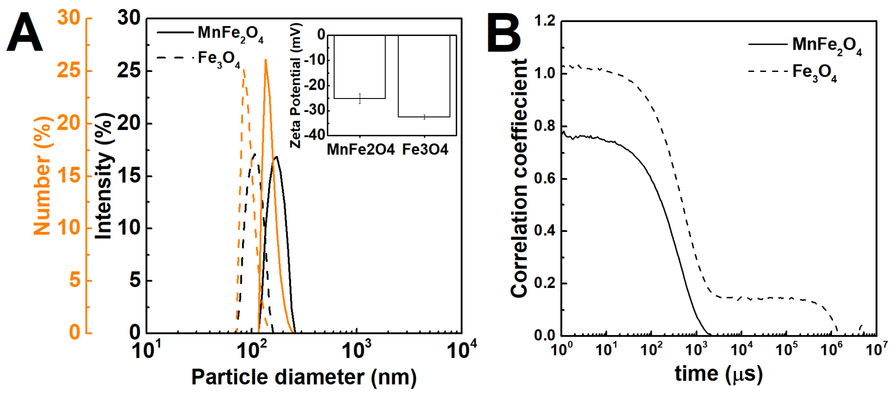

Figure S3. (A) Dynamic light scattering intensity-weighted (black) and number-weighted (orange) distributions of manganese ferrite ($MnFe_2O_4$) and magnetite (Fe_3O_4) nanoparticles, and (B) the respective correlograms. Inset in Figure S3A: Average zeta potential of the $MnFe_2O_4$ and magnetite Fe_3O_4 nanoparticles.

Table S2. Hydrodynamic diameter (D_H), polydispersity and zeta potential of the synthesized magnetic nanoparticles.

Nanoparticle	D_H (nm)	PDI	Zeta potential (mV)
Fe_3O_4	148 ± 1	0.10 ± 0.02	-32.6 ± 0.9
$MnFe_2O_4$	247 ± 15	0.16 ± 0.06	-25.2 ± 1.9

The presence of structural defects, such as non-stoichiometric crystals, vacancies, crystalline structure disorders or lattice distortion, can influence the optical properties of the nanoparticles, leading to the formation of a localized state between the valence band and conduction band. The localized states or defects result in an exponential band tail that is described by the equation:

$$\alpha(\lambda) = \alpha_0 \cdot e^{\frac{E(\lambda)-E_0}{E_U}} \quad \text{(S2)}$$

where, E_U is the Urbach energy, and E_o is the onset of absorption, and can be linearized as:

$$\ln(\alpha) = \ln(\alpha_0) + \frac{E(\lambda) - E_0}{E_U} \quad \text{(S3)}$$

The Urbach energy, E_U, which is reported to be inversely proportional to the amount of defects in the crystal, is calculated from the reciprocal of the slope of ln α vs. photon energy. Additionally, the Urbach theory predicts that α is proportional to 1/E_U, which means that optical transitions are more intense for crystals with smaller amount of defect [1].

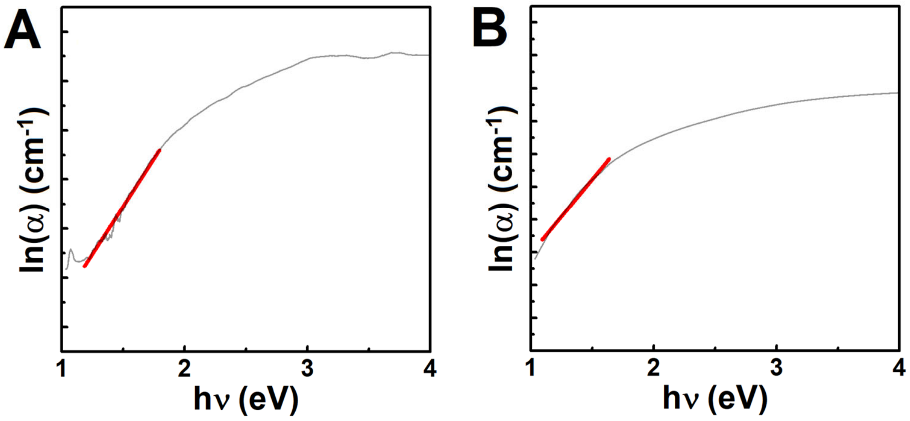

Figure S4. Urbach plots of the (A) Fe_3O_4 and (B) $MnFe_2O_4$ nanoparticles used for the determination of Urbach energy.

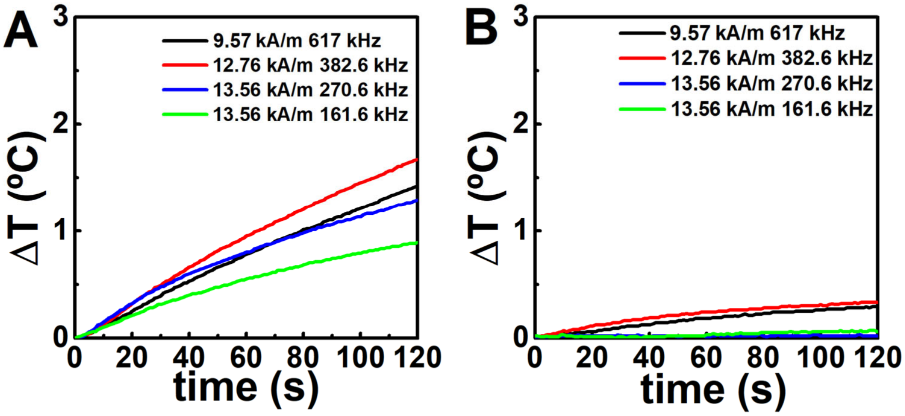

Figure S5. Temperature variation over time of the (A) Fe_3O_4 and (B) $MnFe_2O_4$ nanoparticles at 5 mg/mL upon exposure to an alternating magnetic field with variable intensity and frequency.

Functionalization with citrate and bovine serum albumin

The Fe_3O_4 particles functionalized with BSA were labelled NPs@BSA(x) (where x=0.8, 2.4, and 4.0), corresponding to reaction with 0.8, 2.4, and 4 mg/mL of BSA.

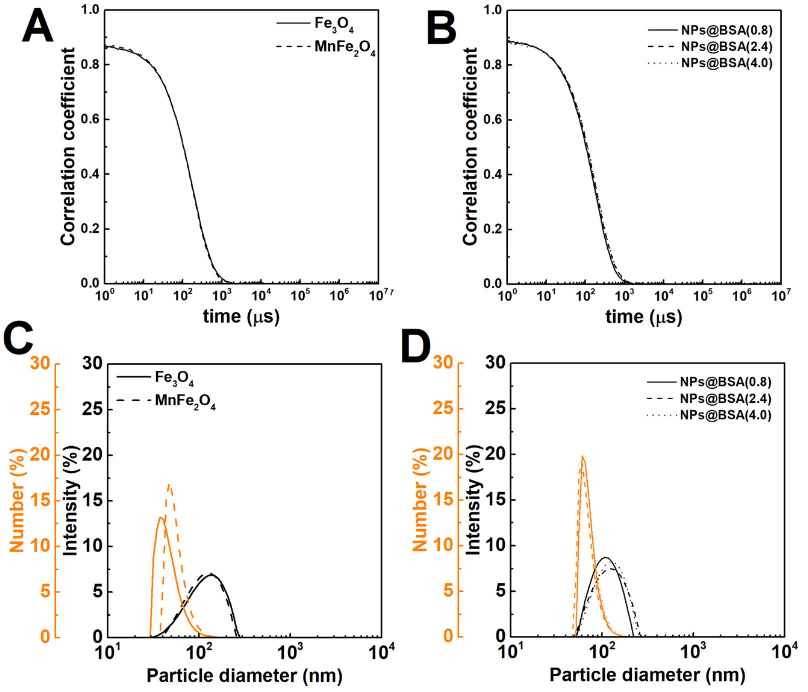

Figure S6. Correlograms of (A) nanoparticles with citrate, and (B) Fe_3O_4 nanoparticles functionalized with BSA, and (C,D) the respective dynamic light scattering intensity-weighted (black) and number-weighted (orange) distributions.

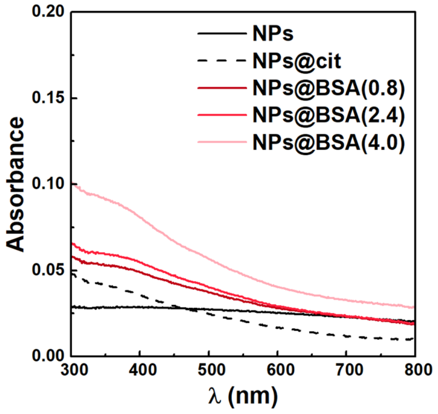

Figure S7. UV-vis absorption spectra of the Fe_3O_4 nanoparticles with and without citrate, and functionalized with BSA at 0.01 mg/mL.

In Figure S7, for the same particle mass concentration, the increasing contribution in the near the infrared region (>700 nm) compared to the NPs@Cit suggested the formation of larger particles upon the functionalization with BSA.

Development of magnetic nanogels

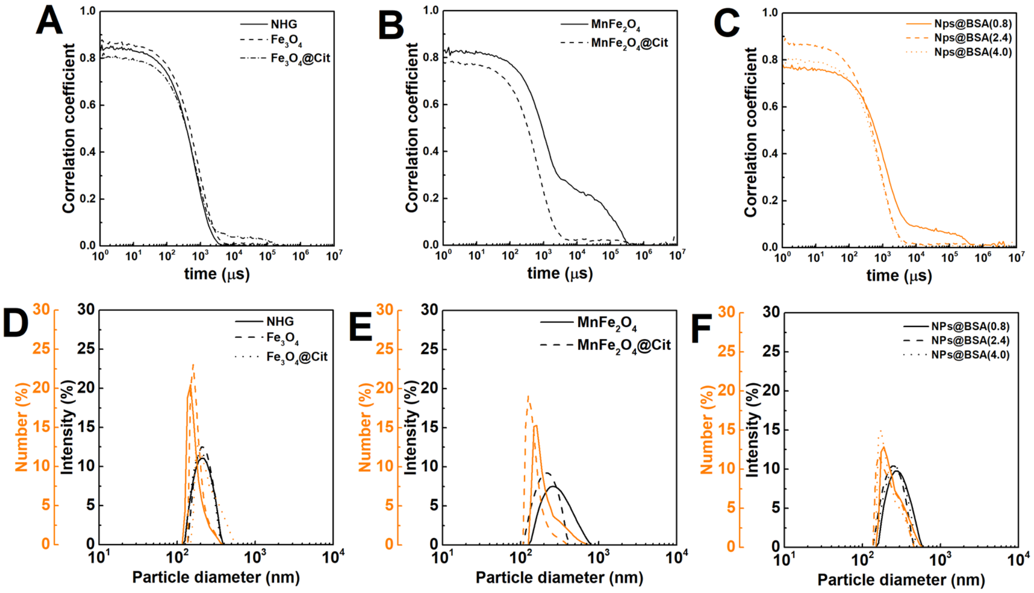

Figure S8. Correlograms of (A) nano-hydrogel (NHG), and magnetic nanogels containing Fe_3O_4 and (B) $MnFe_2O_4$ nanoparticles with and without citrate, (C) Fe_3O_4 nanoparticles functionalized with BSA, and (D-F) the respective dynamic light scattering intensity-weighted (black) and number-weighted (orange) distributions.

Swelling kinetics of magnetic nanogels

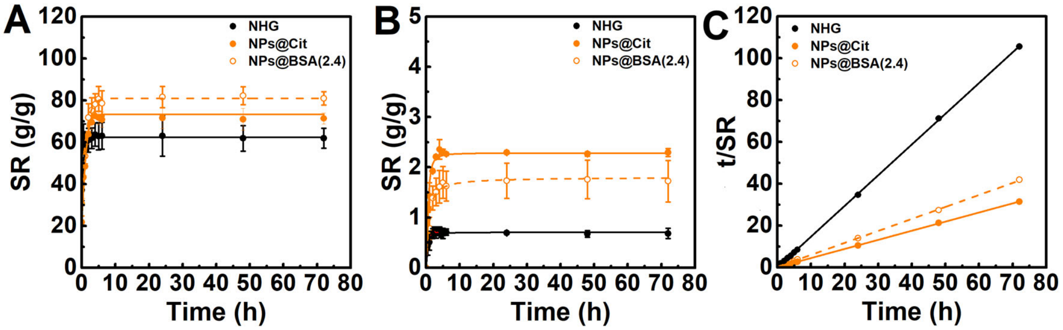

Figure S9. (A) Swelling curves of magnetic nanogels containing citrate- and BSA-functionalized nanoparticles, and nano-hydrogel (NHG) in phosphate buffer 0.1 M pH=7.4. (B) Swelling curves of the gels expressed as $SR = (W_s - W_i)/ W_i$, in which W_s is the weighed nanogel at time t and W_i is the initial weight of the nanogel obtained from the preparation. The profiles were fitted to Gompertz model over the 72 h as a guiding line. (C) Swelling kinetics of gels expressed as t/SR versus time (t) obtained for the swelling curves displayed in Figure S9B.

The swelling assays revealed that the magnetic gels could achieve larger swelling ratio than the nano-hydrogels (Figure S9A). In particular, the gels incorporating BSA-functionalized nanoparticles showcased the highest swelling capability. However, the gels containing citrate-stabilized nanoparticles displayed the largest change in swelling ratio when compared to their initial prepared gel state (Figure S9B).
Considering that the swelling curves correspond to the swelling behavior of the prepared nanogels upon immersion in pH=7.4 buffer, the kinetic analysis of gel swelling was carried out in comparison to this initial gel state. As illustrated in Figure S9C, the swelling kinetics can be accurately described by the second-order kinetic model, $\frac{1}{SR} = \frac{1}{kSR_e{}^2} + \frac{1}{SR_e}t$, in which SR is the swelling ratio at time t, SR_e is the equilibrium swelling, and k is the swelling rate [2]. The obtained parameters indicate that the hydro-nanogels exhibited a faster swelling rate than the magnetic nanogels. For further insight on the mechanism of water diffusion, the Korsmeyer-Peppas diffusion model was fitted to the swelling curves, which parameters are included in Table S3. The value of n lower than 0.5 suggests that water is transported through a diffusion-controlled mechanism, which is advantageous for controlled drug delivery applications.

Table S3. Parameters obtained from the fitting of the Korsmeyer-Peppas to the initial 60% of the swelling curves, and from the fitting of the second-order swelling kinetics model.

	Korsmeyer-Peppas			Second-order swelling kinetics model		
**System**	K_S (h^{-1})	n	R^2	k ($g\ g^{-1}\ h^{-1}$)	SR_{eq}	R^2
**NHG**	0.763	0.25	0.99	44.056	0.681	0.99
**NPs@Cit**	0.607	0.40	0.99	1.943	2.296	0.99
**NPs@BSA**	0.552	0.40	0.99	1.356	1.745	0.99

Electron microscopy images of magnetic nanogels

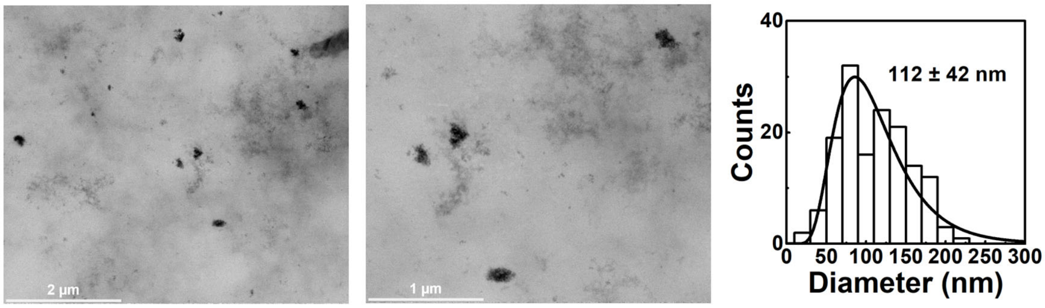

Figure S10. TEM images of the magnetic nanogels containing citrate-stabilized Fe_3O_4 nanoparticles and the respective histogram of the measured agglomerates sizes (N = 150).

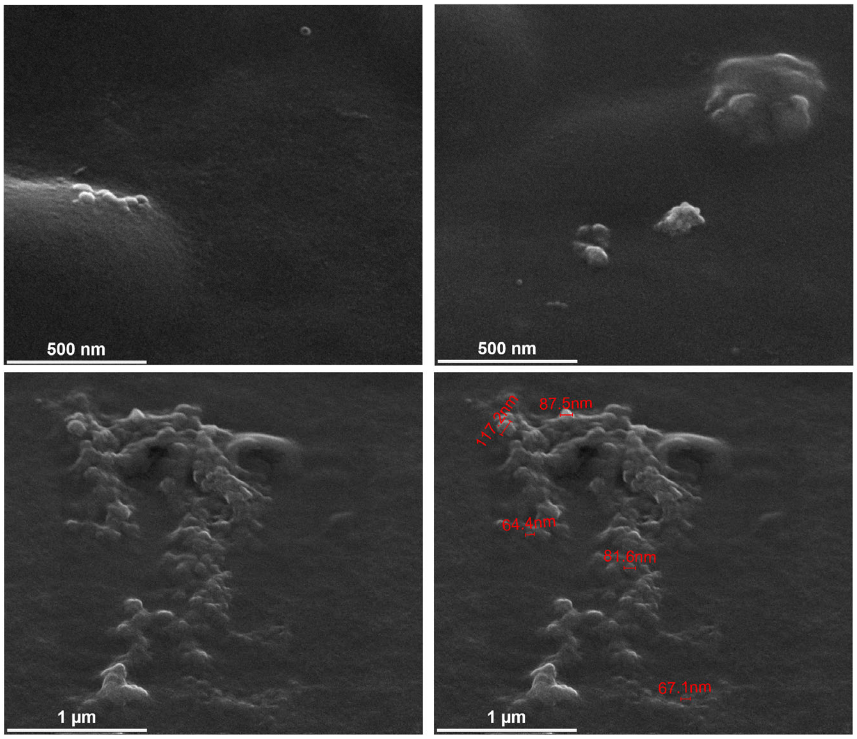

Figure S11. SEM images of the magnetic nanogels containing citrate-stabilized Fe_3O_4 nanoparticles.

The TEM images displayed in Figure S10 provide insights into the nanostructure within the magnetic nanogels. The presence of clusters of magnetic nanoparticles and the obtained size is consistent with the structures observed in SEM images depicted in Figure S11. Thus, the nanogels might be composed of these nanoparticle clusters embedded within the nanogel matrix. Moreover, the images also highlight the possibility of individual nanoparticles coated with nanogel, as evidenced by the presence of smaller structures slightly larger than the magnetic nanoparticles' average size.

Doxorubicin encapsulation effect on hydrodynamic properties

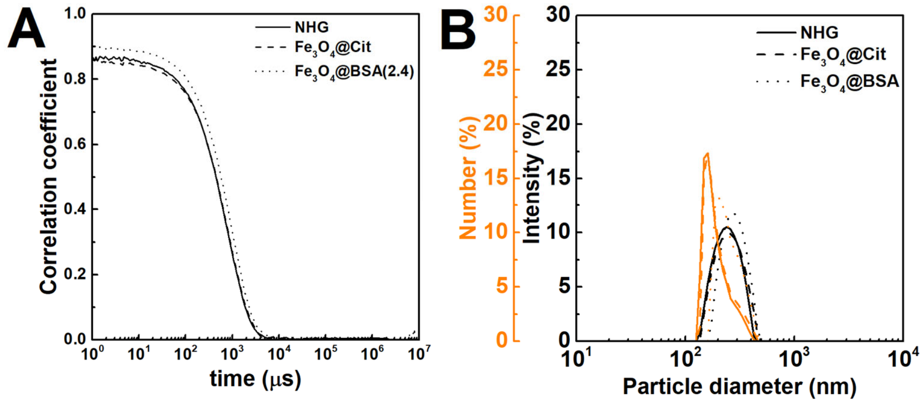

Figure S12. (A) Correlograms and (B) dynamic light scattering intensity-weighted (black) and number-weighted (orange) distributions of doxorubicin-loaded nano-hydrogel (NHG), and magnetic nanogels containing Fe_3O_4 nanoparticles functionalized with citrate or BSA.

Drug release assays

Table S4. Coefficients of determination (R^2) of several fitted models obtained for doxorubicin release profiles in nano-hydrogels, and magnetic nanogels bearing citrate- and BSA-functionalized nanoparticles. The blank spaces correspond to negative coefficients. The mathematical models were fitted to the 76 h release profiles.

System	pH	Stimuli	First-order	Hixson-Crowell	Higuchi	Korsmeyer-Peppas	Gompertz
NHG	6	-	0.85	0.13	0.83	0.99	0.96
	7.4	-	0.77	-	0.67	0.98	0.97
NPs@Cit	6	-	0.83	0.22	0.85	0.99	0.98
		Laser	0.87	0.48	0.94	0.96	0.95
	7.4	-	0.69	-	0.44	0.98	0.98
		Laser	0.83	-	0.79	0.94	0.867
NPs@BSA	6	-	0.83	-	0.78	0.98	0.95
	7.4	-	0.63	-	0.14	0.96	0.95

The Gompertz and Korsmeyer-Peppas models are, respectively, described according to the equations:

$$X_t = X_{max} e^{-ae^{b \log_{10} t}} \quad \text{(S4)}$$

$$\frac{M_t}{M_\infty} = K_s t^n \quad \text{(S5)}$$

in which $\frac{M_t}{M_\infty}$ is the fraction of drug released at time *t*, and K_s is the rate constant. For a spherical geometry, when $n < 0.43$, the release mechanism is diffusion-controlled (Fickian diffusion), $0.43 < n < 0.85$ is an anomalous transport, and $n \geq 0.85$ indicates that the release is mainly driven by swelling or relaxation of network chains (case-II transport) [3,4]. The X_t and X_{max} are the dissolved drug fractions at time t and its maximum, a is a shape parameter and b is the dissolution rate per unit of time.

Table S5. Release coefficients of the Korsmeyer-Peppas and Gompertz models obtained for doxorubicin release profiles in nano-hydrogels, and magnetic nanogels bearing citrate- and BSA-functionalized nanoparticles. The Korsmeyer-Peppas model was fitted to the initial 60% of the drug release profile. The parameter X_{max} of the Gompertz model was fixed at value 1.

			Korsmeyer-Peppas			Gompertz			
**System**	**pH**	**Stimuli**	K_S (h^{-n})	n	R^2	X_{max}	a	b	R^2
NHG	6	-	0.021	0.32	0.99	1	4.02	0.27	0.96
	7.4	-	0.018	0.48	0.99	1	3.94	0.23	0.97
NPs@Cit	6	-	0.019	0.43	0.99	1	4.01	0.28	0.98
		Laser	0.023	0.40	0.99	1	4.19	0.46	0.95
	7.4	-	0.019	0.41	0.99	1	3.84	0.20	0.98
		Laser	0.024	0.43	0.99	1	3.85	0.33	0.87
NPs@BSA	6	-	0.021	0.42	0.99	1	3.93	0.27	0.95
	7.4	-	0.018	0.41	0.99	1	3.89	0.16	0.95

References

1. Bertuit, E.; Benassai, E.; Mériguet, G.; Greneche, J.-M.; Baptiste, B.; Neveu, S.; Wilhelm, C.; Abou-Hassan, A. Structure–Property–Function Relationships of Iron Oxide Multicore Nanoflowers in Magnetic Hyperthermia and Photothermia. *ACS Nano* **2022**, *16*, 271–284, doi:10.1021/acsnano.1c06212.

2. Yavari, N.; Azizian, S. Mixed Diffusion and Relaxation Kinetics Model for Hydrogels Swelling. *J. Mol. Liq.* **2022**, *363*, 119861, doi:10.1016/j.molliq.2022.119861.

3. Ritger, P.L.; Peppas, N.A. A Simple Equation for Description of Solute Release II. Fickian and Anomalous Release from Swellable Devices. *J. Control. Release* **1987**, *5*, 37–42, doi:10.1016/0168-3659(87)90035-6.

4. Dash, S.; Murthy, P.N.; Nath, L.; Chowdhury, P. Kinetic Modeling on Drug Release from Controlled Drug Delivery Systems. *Acta Pol. Pharm. - Drug Res.* **2010**, *67*, 217–223.
